# Nursing Robots Can Reduce Nursing Workload in General Adult Wards: A Two-Phase Study

**DOI:** 10.1155/jonm/9096837

**Published:** 2025-06-04

**Authors:** Yulei Song, Jiaojiao Gao, Yuqing Chen, Jiarui Shi, Xueqing Zhang, Dan Luo, Mengmeng Wang, Ye Wang, Qiongqiong Zang, Zhiyi Pei, Yamei Bai, Guihua Xu

**Affiliations:** School of Nursing, Nanjing University of Chinese Medicine, Nanjing 210023, China

**Keywords:** adult ward, nursing, nursing workload, robotics

## Abstract

**Aim:** This study aims to ascertain the range of nursing care tasks that can be performed by currently available nursing robots in general adult wards, as well as to quantify the extent to which these robots can potentially replace the nursing care workload when implemented.

**Background:** The global shortage of nurses has emerged as a significant societal issue, and nursing robots may be a potential solution to alleviate this crisis. However, there is a dearth of empirical evidence regarding the specific tasks that nursing robots can effectively replace in general adult wards within hospitals and lacking comprehensive reports that quantify how much nursing workload can be reduced by nursing robots.

**Methods:** This research utilizes a two-phase research design with a scoping review and cross-sectional survey, involving the searching and screening of 10 scientific databases to identify nursing robots suitable for deployment in general adult wards within hospital settings. Additionally, the study measures the 24 h nursing workload for all patients across 72 wards in six hospitals located in eastern, central, and western regions of China.

**Results:** 199 studies were included in this scoping review. A total of 26 nursing functions that can be performed by nursing robots in general adult wards were summarized. 7073 hospitalized patients were included in this study. 26 nursing robots have the potential to substitute for 62.37% of the total nursing workload per capita.

**Conclusions:** The use of nursing robots in general adult wards is promising. However, it is worth noting that while nursing programs with the largest share of nursing workload already have nursing robots in use, there are some robots whose development focus and direction need to be adapted to the needs of the clinical nursing workload.

**Implications for Nursing Management:** It is imperative for nursing managers to receive training in order to increase their understanding of robotic nurses and to explore effective and optimized models for managing nursing human resources when robots and nurses collaborate. Simultaneously, the definition and development of core competencies for robotic specialized nurses need to be on the agenda.

## 1. Introduction

The global nursing crisis, acknowledged in 2002, has persisted despite the implementation of effective strategies by the International Council of Nurses (ICN), the World Health Organization (WHO), and national nursing associations worldwide. Regrettably, tangible advancements in addressing nursing shortages have not materialized [1]. Furthermore, the exacerbation of this issue is anticipated due to additional factors including the COVID-19 pandemic, an aging population, and the ongoing attrition of nursing professionals. The State of the World's Nursing 2020 report indicates that the global workforce will experience a deficit of up to 4.6 million nurses by 2030. This scarcity inevitably results in an increased burden on the remaining nursing staff, leading to heightened levels of stress and burnout among nurses [2]. Consequently, job satisfaction diminishes, while turnover intentions rise [3]. Meanwhile, higher nursing workloads and inadequate levels of nursing staffing are also often closely associated with lower quality of nursing care [4] and reduced patient safety [5]. Hence, the presence of persistent nursing shortages will engender a multitude of issues, including inadequate nursing care and diminished nursing staff retention rates, thereby presenting a significant societal predicament for both China and the global community. In order to address this quandary, it is imperative to not only sustain a continuous recruitment of new registered nurses but also to contemplate strategies for enhancing nurses' efficacy and alleviating their workload amidst the constraints of limited nursing personnel, which also poses a challenge for nursing administrators.

The rapid advancement of artificial intelligence technology has led to the emergence of robotics as a promising avenue for addressing this concern. Robots, equipped with complex sensors, transmitters, receivers, and other basic machine components, are autonomous and independent machines that are fully integrated with robot computer software [6]. Due to their intelligent, sensing, and autonomous features [7], the development of nursing robots is increasingly prevalent, aiming to assist nurses with dangerous, heavy, and menial nursing tasks, thereby alleviating their nursing workload and mitigating associated stress. Nursing robots are robots that serve as supplemental healthcare workers in hospitals, older-person care facilities, and at home, and they can perform logistics and laborious physical tasks, combat loneliness and inactivity in the elderly population, or be assigned routine tasks [8]. Based on their designated application scenarios, nursing robots can be classified into three categories: nursing robots for hospitals, nursing robots for nursing homes, and household nursing robots. Initially, the focus of nursing robots was primarily on home use, specifically catering to the needs of the elderly and individuals with disabilities. As early as the 1980s, Handy, a daily life care robot created by Mike Topping, a British company, emerged as a valuable aid for individuals with cerebral palsy, stroke, muscular dystrophy, and injuries resulting from accidents. This robotic technology facilitated the completion of essential daily tasks, including eating and cleaning [9]. Additionally, the German-developed Care-O-bot robot can support the elderly in home activities and monitor their safety, reducing the burden on family caregivers [10]. Nursing robots for nursing homes have proven to be instrumental in delivering vital life support and psycho-social assistance to the elderly. Among these robots, Paro, a seal robot, has gained significant popularity for its involvement in psychiatric care interventions for patients with Alzheimer's disease, earning recognition from numerous nursing professionals [11]. In recent years, Ebola, COVID-19, and other infectious diseases have become pandemic and escalating, and hospital nursing robots are also emerging. To enable healthcare workers to reduce direct contact with patients and diminish their exposure and risk of infection, robots have been designed for sterilizing patient areas, taking throat swabs, and delivering medications and food, among other nursing tasks [12]. And it plays an important role in replacing nursing staff in the prevention, diagnosis, and screening of infectious diseases, as well as in patient care and disease management [13].

Nieto et al. [14] conducted a comprehensive review of 133 nursing robots capable of addressing nursing challenges and identified three predominant technological categories that define the relationships between nurses, robots, and patients: cooperative, collaborative, and supportive. It is indisputable that nursing robots play a supportive and complementary role in the professional duties of nurses. Nevertheless, the significance and potential of nursing robots extend beyond their conventional auxiliary functions, encompassing the prospect of substantial “substitution” in the future. The nursing robot HelpMate, created by Tingley Rubber Corporation in the U.S. [15], and a robot designed by the Massachusetts Institute of Technology (MIT) [16] are all available to replace nurses in performing the specialized nursing tasks required for basic treatment of a patient during a hospital stay. As such, it is clear that the involvement of robots will have a direct impact on nursing practice, and robotics has the potential to serve as a viable alternative for providing mechanistic nursing tasks such as medication dispensing [17]. Most nursing managers also have expressed that robotic nurses can reduce nurses' workload and are beneficial to nurses [18], which could be a way to alleviate the nursing shortage. Existing researches on nursing robots are mainly focused on the development and application of different types of nursing robots or qualitative studies addressing the attitudes and needs of nursing robots, but there are few comprehensive reports that quantitatively analyze how much nursing workload can be reduced by nursing robots.

General adult wards, apart from specialized units such as outpatient clinics, operating rooms, and intensive care units (ICUs), are the areas of the hospital with the highest proportion of patients and nurses, while simultaneously experiencing a severe scarcity of nursing staff [19]. In this context, the utilization of nursing robots holds immense potential for mitigating the nursing workload. Consequently, this study focuses on nursing robots that can directly reduce nursing hours by serving as supplemental nurses in general hospital wards, and it was guided by the following research questions: what nursing tasks can robots, including the ongoing development of prototype nursing robots, supplant nurses in general adult wards? And what magnitude of reduction in nursing workload could be achieved through the implementation of these robots?

## 2. Methods

This study addresses the aforementioned research inquiries through a two-phase study. The first phase involved a scoping review to define which nursing tasks the robot could assist or replace nurses in general adult wards, encompassing ongoing development of prototype nursing robots. In the second phase, a cross-sectional survey of the 24 h nursing workload in general adult wards was conducted to assess the extent of the reduction in nursing workload that would be achieved if existing nursing robots were put into clinical practice.

### 2.1. Literature Scoping Review

This paper utilizes the five-step framework developed by Arksey and O'Malley to conduct a literature scope review [20].

#### 2.1.1. Identifying the Research Question and Identifying Relevant Studies

The search strategy employed in this review involved the utilization of predetermined key search terms: “robot” OR “robotics” OR “robotic systems” OR “intelligent systems” AND “nursing” OR “nurses” OR “caregivers” OR “registered nurses” OR “nursing staff”. A comprehensive search was conducted across four Chinese electronic databases, including CNKI, Wanfang, VIP, and SinoMed, as well as six foreign databases, namely PubMed, Web of Science, CINAHL, ScienceDirect, Springer, and JBI Complete Package Evidence-Based Nursing (Appendix I). The search encompassed various types of literature, including journal articles, dissertations, and patents, published from 1985 to June 6, 2023. Due to user rights, Chinese patents were exclusively searched.

#### 2.1.2. Study Selection and Charting the Data

The inclusion criteria for this review were established based on the Population, Concept, and Context (PCC) principle [21]: (a) Population: registered nurses engaged in clinical nursing. (b) Concept: any invention, observation, or intervention oriented toward nursing robots with a distinct and well-defined nursing function. To be considered relevant for inclusion, the articles must pertain to fulfill the category of nursing robots as defined in this review, i.e., robots that perform nursing tasks in general adult hospital wards by nurse-issued commands, with or without patient involvement, and that can directly reduce nursing work hours. (c) Context: general adult ward of a hospital. Following the completion of the article screening process, information about the title, author, country, time, publication type, robot name, alternative/assistive nursing tasks, and commercial status was extracted. The entire selection and data extraction process was carried out by two research team members, and any disagreements were resolved by consensus, with a third reviewer consulted if necessary.

### 2.2. Nursing Workload Survey

#### 2.2.1. Design and Setting

This section employs a cross-sectional survey design. The sample consisted of patients admitted to general adult wards in China. Patients underwent direct observation to ascertain the nursing programs and hours they received. Employing stratified sampling, China was stratified into East, Central, and West regions, excluding Hong Kong, Taiwan, Macao, and other territories. Two hospitals with a minimum of 1000 beds were then selected from each region. Ultimately, a total of six public tertiary hospitals, willing to participate in the survey, were chosen. Within each hospital, six medical departments (nephrology, gastroenterology, respiratory medicine, endocrinology, neurology, and cardiology) and six surgical departments (general surgery, orthopedic surgery, urology, anorectal surgery, breast surgery, and otorhinolaryngology) were chosen for inclusion in the study. Therefore, this study encompassed 72 wards across 6 hospitals. Survey data were collected from January to December 2016. Prior to participation, informed consent was obtained from both nurses and patients. The study received approval from the Ethics Committee of Hospital of Traditional Chinese Medicine, Jiangsu Province, China (no. 2016NL-049-02).

#### 2.2.2. Sample Size

The primary objective of this study was to furnish descriptive statistics on the average nursing hours worked, obviating the necessity for a specific sample size [22]. To enhance the reliability and generalizability of the findings, 100 cases were investigated in each medical and surgical ward of every hospital, culminating in a comprehensive sample size of 7200. After excluding some invalid data (e.g. data with incomplete information) through manual screening, the effective recovery rate was 98.24%, indicating inclusion of 7073 valid data.

#### 2.2.3. Survey Tools

We used the team-developed WeChat application for data collection and entry for the on-site survey. Patients were surveyed on their demographics, clinical characteristics, 24 h direct nursing programs, frequency, and time required. In order to assess patients' self-care ability and disease severity, the Barthel index (BI) [23] and the simple clinical score (SCS) [24] were utilized. These scales were integrated as spreadsheets within the WeChat application developed by the research team. The measurements of work hours were conducted with utmost precision, down to the second. The single-person nursing program was measured from the beginning of the nurse's item preparation to the end of the final item disposal. In the case of multiperson collaborative nursing programs, the nursing time was calculated by multiplying the measured time by the number of nurses and subsequently dividing it by the number of patients.

#### 2.2.4. Data Collection and Analysis Methods

To ensure the survey's quality, a 1-week pilot workload measurement survey was carried out preceding the formal survey. The aim of this pilot study was to fine-tune the implementation plan for the study. Addressing issues identified during clinical utilization and other functional needs, web technicians were engaged to debug and enhance the WeChat application.

At the commencement of the formal investigation, each hospital set up a survey team, with one project leader, one quality supervisor, and three to four investigators in each department. Before the survey, a unified training and assessment were conducted for all team members. The investigators utilized a WeChat application developed by the team to measure the 24 h nursing hours of patients, and the quality supervisors were responsible for data verification. Subsequently, all data were analyzed with SPSS version 26. Measurement data were expressed as the mean (SD), and count data were described by the rate and percentage. Due to the non-normal distribution of the average 24 h nursing workload, the Mann–Whitney *U* test was used to compare the nursing workload between the medical and surgical wards. A *p* value <  0.05 two-tailed was considered as statistically significant.

## 3. Results

### 3.1. Scoping Review Results

**Figure 1:**
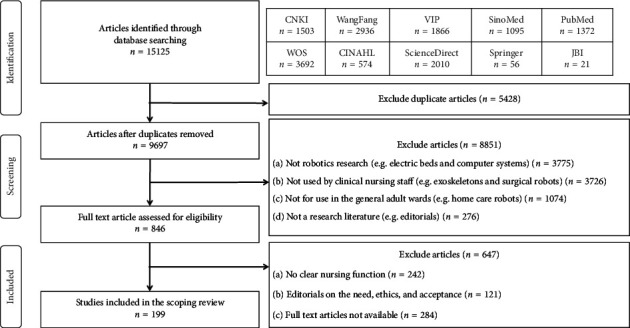
Scoping review screening process.

The screening process is shown in Figure 1. In this paper, a total of 15,125 articles were retrieved from the database. Following the process of deduplication, a total of 5428 papers were excluded. Subsequently, the remaining papers (*n* = 9697) underwent screening at the title and abstract levels, resulting in the exclusion of 8851 papers. Notably, this exclusion encompassed articles that did not align with the specific scope of nursing robotics as defined within the context of this review, such as robotic devices, wearable sensors, electric beds, exoskeletons, surgical robots, and home care robots. There were still 846 articles that needed to be reviewed in full text. Ultimately, 199 studies were included in this scoping review, including 77 Chinese journal papers, 26 foreign language publications, and 96 Chinese patents (Appendix II). These studies were published between 1996 and 2023; 71.9% of the robots were in the design phase, 12.6% were under testing, and only 15.6% of the nursing robots were already in use. Among them, 28 papers (14.1%) reported multiple functions of a particular nursing robot, and there were also several papers describing different stages of development of the same nursing robot (Table 1).

We summarize the 26 nursing functions in Table 2 that can be achieved by nursing robots in existing reports, which are classified into two categories of basic and specialized nursing programs according to the technical difficulty of the nursing programs. Of these, nursing robots could realize a total of 14 basic nursing programs (53.85%), and transfer assist robots had the most studies with 68 reports (34.17%), which was far ahead of other nursing robots. Nursing robots used for delivering medications and other items (N = 45, 22.61%) and for ward rounds (N = 18, 9.05%) were the second and third most frequently reported types, following transfer assist robots. The nursing robot enables 12 (46.15%) specialized nursing programs. Drug configurations for both intravenous (IV) infusions and phlebotomy robots had 12 studies (6.03%), and IV fluid waste disposal, urinary catheterization, and electromagnetic therapy robots were reported in only one study (0.50%).

### 3.2. Nursing Workload Survey Results

#### 3.2.1. Patient Demographics and Clinical Characteristics

A total of 7073 inpatients from 72 wards in 6 hospitals participated in this study, of which 3554 (50.25%) were in medical wards and 3519 (49.75%) were in surgical wards. There were 3652 male inpatients (51.63%) and 3421 female inpatients (48.37%), and the average age was 58.53 ± 18.53 years old. The patients were mostly able to take care of themselves completely in daily life (33.34%) or mildly dependent on the care of others (32.97%), and their condition was not serious (58.63%) (Table 3).

#### 3.2.2. Nursing Workload Results

Table 4 presents a comprehensive overview of the distribution of all nursing programs (Appendix III) and robotically replaceable nursing programs in terms of number and nursing workload in the medical and surgical departments. 7073 patients received a total of 217 direct nursing programs, with an average total nursing hour of 2.32 h/person/24 h (SD = 1.99). There were 40 basic nursing programs with an average nursing hour of 1.30 h/person/24 h (SD = 1.27) and 177 specialized nursing programs with an average nursing hour of 1.02 h/person/24 h (SD = 0.96). A total of 1.44 h/person/24 h (SD = 1.21) is spent on the 26 nursing programs that can be replaced by the nursing robot, accounting for 62.37% of the total work hours. There were 14 basic nursing programs with an average nursing hour of 0.91 h/person/24 h (SD = 0.89) and 12 specialized nursing programs that could be replaced by robots with an average nursing hour of 0.53 h/person/24 h (SD = 0.50). The number of all direct nursing programs and nursing workload received by medical patients was higher than that of surgical patients. Among the 26 nursing programs that could be replaced by nursing robots, except for the absence of electromagnetic therapy programs in surgical wards, the remaining 25 robots could be used in both medical and surgical applications. The nursing robot could replace 14 basic nursing programs in both the medical and surgical departments, with an average of 0.99 h/person/24 h (SD = 0.95) for 3554 medical patients and 0.83 h/person/24 h (SD = 0.82) for 3519 surgical patients.

Table 5 shows the average 24 h nursing workload for 26 robotically replaceable nursing programs in medical and surgical departments during the survey period. Of the 26 nursing programs that could be replaced by robots, ward rounds had the highest nursing workload, followed by psycho-social support. The average time spent on ward rounds per day was 0.53 h/person/24 h (SD = 0.61) for all patients, while 0.58 h/person/24 h (SD = 0.67) was required for patients in the medical wards and 0.48 h/person/24 h (SD = 0.55) in the surgical wards. There was a statistical difference between the medical and surgical wards in several nursing tasks. Only in five nursing programs, disinfection of wards (*p*=0.057), health education (*p*=0.128), expectoration (*p*=0.092), take body temperature and pulse (*p*=0.062), and take blood oxygen saturation (*p*=0.066), the differences were not statistically significant.

## 4. Discussion

This study found through a scoping review that robots, including a prototype nursing robot under development, could potentially supplant or assist nursing personnel in general adult wards with 26 nursing tasks, such as ward rounds, transfer assistance, deliver medications, and other items. Integrating these robotic technologies could potentially reduce nursing work hours by 62.37%.

This study found through a scoping review that the development of nursing robots for general adult wards in hospitals occurred slightly later than the emergence of geriatric care robots for home or nursing home use. The first nursing robots in general adult wards in hospitals were developed by TRC, founded by Joseph F·Engelberger, the father of robotics, and sold in 1990 as “nurse's aides” for delivering medications and other items in hospital wards. Immediately after, Japan also designed transfer assist robots aimed at assisting nurses in lifting and repositioning bedridden patients in ward settings. Following the year 2000, the nursing robotic sector in both the United Kingdom and China began to take off, and robots for phlebotomy, ward rounds, and ward sterilization were developed one after another. In 2010, the advancement of hospital general ward nursing robots witnessed a significant acceleration, with an average of four novel robot designs introduced annually. Notably, the year 2016 marked a turning point, as the open AI ecosystem with robotics as an important branch was listed as one of the ten most important emerging technologies by the World Economic Forum [25], and the quantity of nursing robots developed began to exhibit exponential growth on a yearly basis. In recent years, globally, the trend of population aging has deepened [26]. The popularity of COVID-19 has further exposed the pressure on the traditional healthcare system and the increasing demand for nursing care. Nursing robotics development achieved an unprecedented leap. Remarkably, in the three years starting in 2020, the quantity and functionality of hospital general ward nursing robots under development have nearly equaled the cumulative total of previous years. Similar to the findings of Ohneberg's scoping review on assistive robots for geriatric care [27], this study found that 71.9% of nursing robots developed for general hospital wards are still in the design phase, 12.6% are in testing, and only 15.6% are in clinical use. The integration of nursing robots into clinical practice involves several stages, including product development, formulation of marketing strategies, and evaluation of implementation effectiveness. This process is impeded by various factors, such as socioeconomics, implementation results from implementation tests, and ethical considerations [28]. In order to prevent the nursing robots that fail to adhere to established ethical guidelines or fail to meet user expectations and to minimize the burden on nurses in maintaining continuous robot operation [29], longer time periods will be used to test the performance and quality of nursing robots. It is apparent that there is still a long way to go to popularize and promote nursing robots.

This study determined that current reports on the utilization of nursing robots within general hospital wards demonstrate that they can realize 26 nursing functions. The quantity of basic and specialized nursing tasks that can be substituted does not significantly vary, but from the number of developments for each function, most studies endeavors primarily concentrate on the development of basic nursing functions. Among these, transfer-assisted nursing robots have received the most attention in relevant studies, followed by robots involved in delivery of medications or other items and ward rounds. These robots are straightforward in function, rendering their development less arduous and expensive, yet they can replace a greater amount of nursing workload and significantly enhance efficiency. They are capable of supplanting nurses' lifting maneuvers during transfers, leading to improved efficiency, safety, and patient comfort [30], as well as reducing the nurses' physical strain and occupational hazards [31]. Or they can navigate predetermined paths, evade obstacles, and monitor the patient's condition more accurately through features such as face recognition and data recognition [32]. The findings of the cross-sectional survey further indicate that ward rounds constitute the most demanding nursing tasks for medical and surgical nurses, accounting for 36.68% of the total nursing hours per capita for all patients. Hence, nursing robots that replace nurses in these basic nursing tasks can greatly reduce the time and physical exertion of nurses. By relieving nurses from repetitive and complicated basic nursing work, this approach enables them to concentrate their efforts on specialized tasks, thereby enhancing the quality of patient care [33] and bolstering nurses' professional identity, ultimately reducing the willingness to leave [34]. It is worth noting that transfer assistance, while occupying a mere 0.2% of a nurse's nursing workload per day, is just as indispensable as other robots as an important means of mitigating occupational injuries among nurses. Therefore, given the availability of adequate robot designs both domestically and internationally, research and development organizations or technology companies should actively redirect their attention towards the testing and general promotion of transfer-assisted robots. In addition to this, in the nursing workload survey of basic nursing care, psycho-social support is a task that has the second highest total nursing work hours per patient among 26 nursing programs. This demonstrates the considerable potential of nursing robots within this domain. Due to the cost-effectiveness and well-established utilization of machine-assisted learning technology [35], the advancement of chat nursing robots for psycho-social support and health promotion has gained significant momentum. The earliest socially assistive robots were designed for demented elderly people in nursing homes, taking into account both emotion and cognition [11]. Now the psycho-social support robots in children's and adult wards are more focused on emotions. Nurses can use them to foster positive emotions among hospitalized patients and alleviate negative emotions, including anxiety and depression. However, during the course of the scoping review, we also found that the utilization of psycho-social support robots in adult hospital wards is relatively limited in comparison to their usage in nursing homes and children's wards. Presently, the emergence of ChatGPT, an advanced language model developed by OpenAI, presents an opportunity for these robots to engage in more intelligent and fluent communication [36]. Integrating it rationally into nursing robots designed for psycho-social support or health promotion will also reduce more nursing workload for nurses and therefore warrants further exploration and development.

In contrast to the considerable progress made in the development of robots for basic nursing care, the advancement of robots for specialized nursing care, particularly in foreign countries, remains limited, as evidenced by a mere five reports. Within China, research efforts have primarily focused on the application of robotics in IV drug dispensing, phlebotomy, and vital sign monitoring. Previous studies have shown that, compared to manual operations, nursing robots perform IV drug dispensing with a significantly higher quality of flushing and dispensing, and the incidence of occupational injuries and the work intensity of nurses are significantly reduced [37]. The success rate of venipuncture also exhibited a notable increase, accompanied by a decrease in patient discomfort and adverse occurrences [38]. The results of the field survey also showed that a significant portion of the daily nursing workload is attributed to the tasks of monitoring vital signs and administering IV fluids. In addition, nurses also allocate substantial time to specialized nursing duties, including IV infusion, infusion replacement, and IVFWD. However, the testing and implementation of nursing robots in these areas are scarce, with only one report available on the development of an IVFWD robot, which is still in its early stages. This phenomenon may be attributed to the inherent nature of specialized nursing programs, which predominantly involve invasive procedures and direct interaction with patients, thereby encompassing greater intricacy and diversity. Consequently, the development of such programs becomes arduous. Simultaneously, the imperative adherence to legal and ethical standards mandates the preservation of patient privacy and safety in healthcare settings [39]. Therefore, nursing robots must undergo meticulous testing and validation processes, leading to escalated costs and prolonged durations for their development and widespread implementation.

Interestingly, in our scoping review, it was discovered that 28 publications reported on composite nursing robots. Similar to the reports on single nursing functions, the composite nursing robot primarily relies on the integration of various basic nursing tasks. The combination of functions in these robots can be broadly categorized into three groups. The first category involves the amalgamation of multiple nursing functions that operate on the same principle. For instance, ward rounds, delivery of medication or other items, and ward disinfection are all care items that are executed by navigating obstacles to reach the ward. Admission introduction, discharge guidance, and health education are all nursing programs that are carried out by communicating and interacting with patients through voice. The second type is a combination of multiple nursing functions of the same type that are interrelated, such as temperature measurement often combined with blood pressure and pulse measurement in research and development. The third type involves a fusion of first two types of functional combinations, such as utilizing obstacle avoidance to navigate to the ward or employing voice communication with the patient, coupled with other functions such as vital sign monitoring. In the future, the development of “multifunctional” nursing robots could potentially serve as a means to reduce the cost of nursing robot development and improve the efficiency of both research and development.

According to the findings of the nursing workload survey, it is evident that the workload of the 26 nursing programs, which can potentially be substituted by nursing robots, accounts for 62.37% of the total work hours per capita. It is clear that the main functions of nursing robots currently being developed cover almost all nursing programs that account for a large proportion of the clinical workload. If the robot is really put into clinical use, it will supplant a substantial portion of the nursing workload, and the application prospect is bright. In our findings, with the exception of the electromagnetic therapy program, which is specific to medical department, all of the 25 nursing machines can be applied in both medical and surgical departments. Compared to surgical wards, nursing robots have the potential to assume a greater portion of nursing workload in medical wards. This is attributed to the distinct characteristics inherent to each department, with surgical wards being dominated by surgical treatment. Thus, the nursing workload that can be potentially delegated to nursing robots in nursing programs such as preoperative education and psycho-social support, postoperative replacement of infusion fluids, and monitoring of vital signs, demonstrates a higher potential for substitution in surgical units.

Nursing robots are a broad concept, presenting a diversified field because of different elements such as the robot type, operator, and application scenarios. While the primary objective of this study was to investigate the potential of nursing robots in reducing nursing work hours in general adult wards, we have also found in our research that robots also have a much broader application in nursing decision making and nurse training within such wards in hospitals. The small robots created by MIT and SoftBank Robotics can assist nurses make simple clinical decisions by leveraging machine learning algorithms, thus diverting their attention to more difficult or complex decisions [40]. Furthermore, numerous robots have been developed with the capability to replicate authentic symptoms or assume the persona of a patient, thereby facilitating the training and evaluation of nurses and nursing students in specialized proficiencies [41]. While these robots are unable to serve as nursing staff to execute nursing duties and consequently alleviate the nursing workload directly, they have the potential to enhance the efficiency of nurses' work and the quality of their services. This contributes to the sustainable advancement of the nursing profession, warranting further investigation and dissemination. Finally, we would like to emphasize that the future utilization of nursing robots should transcend mere automation and efficiency improvement, instead of focusing on establishing a harmonious ecosystem of collaboration between humans and robots. Within this framework, robots would not only be capable of executing monotonous, perilous, or labor-intensive nursing duties but would also possess the ability to intelligently learn and adapt, thereby enhancing productivity and fostering innovation in conjunction with nurses. It is crucial to underscore that robots serve solely as supplementary tools, while nurses remain the central figures in the nursing profession [14]. Nurses must consistently monitor the advancements in the robotic technology to ensure that nursing can effectively uphold the universal standard of humanistic care within the new system. Simultaneously, nursing management, education, and research should change around the differentiated needs of professional nursing practice.

## 5. Conclusions

This paper provides a scoping review and cross-sectional survey to clarify that robots are able to replace 26 nursing tasks in general adult wards in hospitals, and that if existing nursing robots were put into use in the wards, it would reduce 62.37% of total nursing workload per capita. While nursing robots are currently in development for high-workload nursing programs, it is important to acknowledge that certain robots' developments may not align with the requirements of clinical nursing. Therefore, future advancements in the robot technology should take into account clinical realities, nurse needs, and robot performance in order to minimize nursing workload with maximum efficiency.

### 5.1. Limitations

The scope review of this paper was searched using the keywords “nursing” and “robotics.” Despite the broad inclusion criteria, there may have been limitations in terms of language restrictions (Chinese and English), which may not have covered important studies written in other languages. Because of the lack of user privileges, only Chinese-language patents were searched in the scope review of this paper, and most of the included studies were from China, which limits the generalizability of our conclusions.

## 6. Implication for Nursing Management

This study presents compelling evidence supporting the notion that the implementation of nursing robots holds potential for alleviating the burden on nursing staff. Specifically, it delineates the specific nursing tasks within adult wards that can be effectively undertaken by robots, along with the associated reduction in nursing workload. Consequently, this research offers valuable insights for guiding the future advancement and integration of nursing robots within clinical settings.

Nursing managers must demonstrate responsiveness to technological advancements and promote awareness of robotic nurses through comprehensive training programs. It is imperative for them to actively explore efficient and refined models of human resource management in the context of collaborative work between robots and nurses. The definition and development of core competencies for robotic specialized nurses need to be on the agenda. Simultaneously, it is critically important to address patient safety concerns and ethical considerations associated with the utilization of robots.

## Figures and Tables

**Table 1:** Characterization and distribution of nursing robots in the clinic.

Characterization	*N*	In percent (%)
*Time*		
Before 2000	3	1.5
2001–2005	2	1.0
2006–2010	7	3.5
2011–2015	19	9.5
2016–2020	74	37.2
After 2020	94	47.2

*Publication type*		
Journal articles in Chinese	77	38.7
Journal articles in foreign languages	26	13.1
Chinese patents	96	48.2

*State of the development in robotics*		
In design	143	71.9
Under test	25	12.6
In application	31	15.6

*Number of nursing functions*		
Single function	171	85.9
Multiple functions	28	14.1

**Table 2:** Nursing programs of nursing robots in the clinic.

Nursing programs	Number of journal articles in Chinese	Number of Chinese patents	Number of journal articles in foreign languages	Total	In percent (%)
Basic nursing programs	65	83	25	173	
Bath assistance	0	0	1	1	0.50
Meal assistance	1	0	0	1	0.50
Roll-over assistance	2	5	0	7	3.52
Transfer assistance	21	32	15	68	34.17
Urinary and fecal care	2	3	0	5	2.51
Turn over and pat on the back	0	1	0	1	0.50
Psycho-social support	0	2	4	6	3.02
Health education	3	3	0	6	3.02
Admission guidance	4	1	0	5	2.51
Discharge guidance	1	0	0	1	0.50
Preoperative education	1	0	0	1	0.50
Deliver medications and other items	13	29	3	45	22.61
Disinfection of wards	6	1	1	8	4.02
Ward round	11	6	1	18	9.05
Specialized nursing programs	28	31	5	64	
Drug configurations for intravenous infusions	10	2	0	12	6.03
Drug configurations for other routes of administration	1	2	0	3	1.51
Intravenous infusion	2	5	0	7	3.52
Replacement of infusion vials	1	0	1	2	1.01
Intravenous fluid waste disposal (IVFWD)	1	0	0	1	0.50
Phlebotomy	8	4	0	12	6.03
Take body temperature	1	7	2	10	5.03
Take blood pressure and pulse	1	7	2	10	5.03
Take blood oxygen saturation	1	0	0	1	0.50
Electromagnetic therapy	0	1	0	1	0.50
Expectoration	2	2	0	4	2.01
Urinary catheterization	0	1	0	1	0.50

**Table 3:** Demographic and clinical characteristics of participants (*N* = 7073).

Variables	*N*	In percent (%)
*Department*		
Nephrology	587	8.30
Gastroenterology	596	8.43
Respiratory medicine	499	7.05
Endocrinology	596	8.43
Neurology	591	8.36
Cardiology	685	9.68
General surgery	780	11.03
Orthopedic surgery	778	11.00
Urological surgery	484	6.84
Anorectal surgery	497	7.03
Breast surgery	397	5.61
Otorhinolaryngology	583	8.24

*Gender*		
Male	3652	51.63
Female	3421	48.37

*Age*		
35 years and below	971	13.73
36–45 years	749	10.59
46–55 years	1205	17.04
More than 55 years	4148	58.65

*Rating scale for self-care ability*		
Fully dependent on others' care	1397	19.75
Mostly dependent on others' care	986	13.94
Mildly dependent on others' care	2332	32.97
Not dependent on others' care	2358	33.34

*Rating scale for disease severity*		
Not serious	4147	58.63
Slightly serious	1385	19.58
Moderately serious	742	10.49
Heavily serious	705	9.97
Extremely serious	94	1.33

**Table 4:** Profiles of the nursing program numbers and nursing workload.

Departments and nursing programs	Basic nursing programs				Specialized nursing programs				Total			
	*N*	Mean (min)	SD (min)	In percent (%)	*N*	Mean (min)	SD (min)	In percent (%)	*N*	Mean (min)	SD (min)	In percent (%)
All nursing programs in all departments	40	77.91	76.39	56.07	177	61.04	57.79	43.93	217	138.96	119.57	100.00
Robotically replaceable nursing programs in all departments	14	54.69	53.31	39.36	12	31.98	30.03	23.02	26	86.67	72.78	62.37
All nursing programs in medical departments	40	83.33	84.41	57.21	149	62.32	56.78	42.79	189	145.65	125.92	100.00
Robotically replaceable nursing programs in medical departments	14	59.53	56.83	40.87	12	32.02	25.28	21.98	26	91.55	73.69	62.85
All nursing programs in surgical departments	39	72.44	66.90	54.80	136	59.75	58.77	45.20	175	132.19	112.40	100.00
Robotically replaceable nursing programs in surgical departments	14	49.80	49.02	37.68	11	31.94	34.17	24.16	25	81.75	71.52	61.84

**Table 5:** Average 24 h direct nursing workload per person.

Nursing programs	All patients (*N* = 7073)			Medical patients (*N* = 3554)			Surgical patients (*N* = 3519)			*p*
	Mean (min)	SD (min)	In percent (%)	Mean (min)	SD (min)	In percent (%)	Mean (min)	SD (min)	In percent (%)	
Basic nursing programs	54.69	53.31	63.10	59.53	56.83	65.02	49.80	49.02	60.93	< 0.001^∗∗^
Bath assistance	0.67	3.71	0.77	0.85	4.63	0.93	0.49	2.43	0.60	0.022^∗^
Meal assistance	0.52	4.83	0.60	0.74	5.85	0.81	0.30	3.49	0.37	< 0.001^∗∗^
Roll-over assistance	2.78	10.29	3.21	2.59	11.23	2.83	2.96	9.24	3.63	< 0.001^∗∗^
Transfer assistance	0.29	1.97	0.34	0.32	2.32	0.35	0.27	1.52	0.33	0.003^∗^
Urinary and fecal care	2.14	7.44	2.47	2.61	9.18	2.85	1.67	5.09	2.05	0.001^∗∗^
Turn over and pat on the back	1.67	8.69	1.93	1.78	9.74	1.94	1.57	7.48	1.92	< 0.001^∗∗^
Psycho-social support	8.16	10.09	9.42	7.98	8.62	8.72	8.35	11.38	10.21	0.044^∗^
Health education	0.02	0.83	0.03	0.02	0.93	0.03	0.02	0.70	0.03	0.128
Admission guidance	2.27	4.81	2.62	2.78	5.27	3.04	1.75	4.23	2.14	< 0.001^∗∗^
Discharge guidance	0.91	2.85	1.05	1.03	3.04	1.13	0.78	2.65	0.96	< 0.001^∗∗^
Preoperative education	0.45	2.29	0.52	0.07	0.70	0.07	0.84	3.13	1.03	< 0.001^∗∗^
Deliver medications and other items	1.90	4.18	2.19	2.70	4.75	2.94	1.10	3.34	1.35	< 0.001^∗∗^
Disinfection of wards	1.10	5.36	1.27	1.47	6.32	1.60	0.73	4.15	0.90	0.057
Ward round	31.79	36.73	36.68	34.59	39.98	37.78	28.96	32.89	35.43	< 0.001^∗∗^
Specialized nursing programs	31.98	30.03	36.90	32.02	25.28	34.98	31.94	34.17	39.08	< 0.001^∗∗^
Drug configurations for intravenous infusions	2.19	6.28	2.53	2.26	5.17	2.47	2.12	7.22	2.59	< 0.001^∗∗^
Drug configurations for other routes of administration	1.61	4.91	1.86	1.56	4.92	1.70	1.67	4.90	2.04	< 0.001^∗∗^
Intravenous infusion	4.90	6.49	5.65	5.37	5.79	5.86	4.42	7.10	5.41	< 0.001^∗∗^
Replacement of infusion vials	5.38	13.88	6.21	3.96	6.23	4.32	6.82	18.55	8.34	< 0.001^∗∗^
Intravenous fluid waste disposal (IVFWD)	2.14	2.27	2.46	2.27	2.31	2.48	2.00	2.22	2.45	< 0.001^∗∗^
Phlebotomy	1.77	3.32	2.04	2.16	3.98	2.36	1.38	2.40	1.68	< 0.001^∗∗^
Take blood pressure	5.64	8.95	6.50	5.93	7.81	6.48	5.34	9.96	6.54	< 0.001^∗∗^
Take body temperature and pulse	7.60	11.42	8.76	7.81	11.51	8.53	7.38	11.34	9.03	0.062
Take blood oxygen saturation	0.27	3.12	0.31	0.23	2.68	0.25	0.32	3.50	0.39	0.066
Electromagnetic therapy	0.03	0.65	0.03	0.06	0.92	0.06	0.00	0.00	0.00	< 0.001^∗∗^
Expectoration	0.29	3.33	0.34	0.26	3.03	0.29	0.32	3.62	0.40	0.092
Urinary catheterization	0.18	1.49	0.20	0.17	1.61	0.18	0.19	1.36	0.23	0.003^∗^
Total	86.67	72.78	100.00	91.55	73.69	100.00	81.75	71.52	100.00	< 0.001^∗∗^

^∗^Significant *p* value 0.05.

^∗∗^Significant *p* value 0.001.

## Data Availability

The data that support the findings of this study are divided into two main sections. The data from the scoping review are presented in the supporting information to this article. The data from the cross-sectional survey are not publicly available due to privacy and ethical restrictions and are available on request from the corresponding author.

## Supporting Information

Please see Appendices I-II for the search terms and included literature from the scoping review and Appendix III for the names of all nursing programs in the cross-sectional survey.
