# Supplementary material for: Nursing Robots Can Reduce Nursing Workload in General Adult Wards: A Two-Phase Study
Source: J Nurs Manag. 2025 Jun 4;2025:9096837. doi: 10.1155/jonm/9096837 (PMC12158590; doi:10.1155/jonm/9096837)
Supplement: Supporting Information — Additional supporting information can be found online in the Supporting Information section. [file 9096837.f1.pdf]

Appendix I. Database searching

Database until 2023	Search terms
**CNKI**	(Subject = (Robot * Nursing) + (Robot * Nurse)) OR (Title = (Robot * Nursing) + (Robot * Nurse))
**WANFANG**	(Chinese-English expansion & subject line expansion): (Subject:(Robotics) and Subject:(Nursing OR Nurse)) and Date:*-2023
**VIP**	(Any Fields = Robotics AND (Any Fields = Nursing OR Any Fields = Nurse))
**SinoMed**	"Robotics"[All Fields:Intelligent] AND("Nursing"[All Fields:Intelligent] OR "Nurse"[All Fields:Intelligent])
**PubMed**	((((Robotics[MeSH Terms]) OR (Robot)) OR (robotic systems)) OR (smart systems)) AND ((((((Nursing[MeSH Terms]) OR (Nurses[MeSH Terms])) OR (Nurses)) OR (Registered Nurses)) OR (Nursing Personnel)) OR (caregivers))
**WOS**	1 ((((TS=(Robotics)) OR TS=(Robot))) OR TS=(robotic systems)) OR TS=(smart systems)<br>2 (((((TS=(Nursing)) OR TS=(Nurses)) OR TS=(Nurse)) OR TS=(Registered Nurses)) OR TS=(Nursing Personnel)) OR TS=(caregivers)

	(#1) AND #2
**CINAHL**	S1 SU Robotics OR SU Robot OR SU robotic systems OR SU smart systems<br>S2 SU Nursing OR SU Nurses OR SU Nurse OR SU Registered Nurses OR SU Nursing Personnel OR SU caregivers<br>S3 S1 AND S2
**ScienceDirect**	("robotics" OR "robot" OR "robotic systems" OR "smart systems") AND ("Nursing" OR "Nurse" OR "Registered Nurses" OR "Nursing Personnel" OR "caregivers")
**Springer**	(robotics AND OR AND robot AND OR AND robotic AND systems AND OR AND smart AND systems) AND AND AND (Nursing AND OR AND Nurse AND OR AND Registered AND Nurses AND OR AND Nursing AND Personnel AND OR AND caregivers)
**JBI**	robotics

Appendix II. Summary of selected articles

Table 1. Summary of selected articles

Study reference	Country	Publication type	Robot name	Alternative/Assistible nursing tasks	Commercial status
Baisch et al., 2018	German	Journal articles in foreign languages	Emotional Robot	Psycho-social support	in application
Blaauw et al., 2021	USA	Journal articles in foreign languages	Robotic-Assisted Transfer Device	Transfer assistance	in design
Brinkmann et al., 2022	German	Journal articles in foreign languages	Collaborative Robotic	Transfer assistance	in design
Brunson, Megan, 2020	USA	Journal articles in foreign languages	Moxi	Deliver medications and other items; Deliver medications and other items	in application
Cai Tingting	China	Journal articles	Admission Promotion Robot	Admission guidance	in application

& Huang Xiaoli, 2021		in Chinese			
Cao Yaofeng, 2020	China	Chinese Patent	Automated Drug Dispensing Robot	Drug configurations for other routes of administration	in design
Cao Yu et al., 2022	China	Journal articles in Chinese	Healthcare Robot	Deliver medications and other items	in application
Chao Wang et al., 2022	China	Chinese Patent	Self-Propelled Nursing Robot	Transfer assistance	in design
Chen Guiliang et al., 2018	China	Chinese Patent	Transportation Nursing Robot	Transfer assistance	in design
Chen Kangyu, 2021	China	Journal articles in Chinese	Ward Round Robot	Ward round	under test
Chen Mingjie, 20	UK	Journal articles in Chinese	Intravenous Blood Collection Robot	Phlebotomy	in design

03					
Chen Xinyu et al., 2021	China	Journal articles in Chinese	Urinary and Fecal Care Robot	Urinary and fecal care	in design
Chen Yu et al., 2021	China	Journal articles in Chinese	Medical Blood Collection Robot	Phlebotomy	in design
Chen Zhen et al., 2021	China	Journal articles in Chinese	Medical Robot	Admission guidance; Health education; Take body temperature; Take blood pressure; Take blood oxygen saturation	in application
Chen Zhengqi, 2022	China	Chinese Patent	Nursing Robot	Deliver medications and other items	in design
Chen Zhigang et al., 2020	China	Journal articles in Chinese	Automated Venous Blood Collection Robot	Phlebotomy	in application
Chia-Hao Wu, 2020	China	Journal articles in Chinese	Transport Robot	Transfer assistance	in design
Chunhui	China	Chinese Patent	Body Lifting Robot	Transfer assistance	in design

Yin et al., 2021					
Dai Lingyu et al., 2022	China	Journal articles in Chinese	Intelligent Dispensing Robot	Drug configurations for intravenous infusions	in application
Deng Jieru & Cai Xuepeng, 2019	China	Journal articles in Chinese	Intelligent Infusion Assist Robot	Intravenous infusion	in design
Dong Lili et al., 2021	China	Journal articles in Chinese	Automated Venipuncture Robot	Intravenous infusion	in application
Du Fanjing & Fan Jianan, 2018	China	Chinese Patent	Prevention Drug Delivery Error Robot	Deliver medications and other items	in design
Fa Mou, 1996	China	Journal articles in Chinese	Nurse Assistant Robot	Deliver medications and other items	in application
Fang Xiaonan, 20	China	Journal articles in Chinese	Magic Nurse	Phlebotomy	in design

21					
Farkh et al., 2021	Saudi Arabia	Journal articles in foreign languages	Autonomous Robot	Deliver medications and other items	in design
Fauteux , 2021	USA	Journal articles in foreign languages	Vital Signs Measuring Robot	Take body temperature; Take blood pressure and pulse	in application
Fu Suliang et al., 2021	China	Chinese Patent	Bladder Flushing Fluid Delivery Robot	Deliver medications and other items	in design
Fu Xueqi, 2014	China	Journal articles in Chinese	Infusion Dispensing Robot	Drug configurations for intravenous infusions	in design
Fu Yuxing, 2022	China	Journal articles in Chinese	Multi-Arm Collaborative Cradle Care Robot	Transfer assistance	under test
Gao Fangzheng et al., 2021	China	Chinese Patent	Intelligent Round Robot	Take body temperature; Take blood pressure and pulse; Deliver medications and other items	in design

Gao Xin, 2012	China	Journal articles in Chinese	Ward Round Robot	Ward round	in design
Ge Qiuju et al., 2019	China	Chinese Patent	Intelligent Robot	Take body temperature; Take blood pressure and pulse; Psycho-social support	in design
Gibson et al., 2017	USA	Journal articles in foreign languages	517 - Implementation of Xenon Ultraviolet-C Disinfection Robot	Disinfection of wards	in application
Gong Fanghua et al., 2021	China	Chinese Patent	Intravenous Infusion Robot	Replacement of infusion vials	in design
Greenhalgh et al., 2019	USA	Journal articles in foreign languages	Assisted Transfer Robot	Transfer assistance	in design
Greenhalgh et al., 2022	USA	Journal articles in foreign languages	Assisted Transfer Robot	Transfer assistance	under test
Greenhalgh	USA	Journal articles	Assisted Transfer Robot	Transfer assistance	under test

et al., 2022		in foreign languages			
Greenhalgh et al., 2023	USA	Journal articles in foreign languages	Assisted Transfer Robot	Transfer assistance	under test
Gu Lizhi et al., 2020	China	Chinese Patent	Turnover Care Robot	Roll-over assistance	in design
Gu Qifang, 2018	China	Journal articles in Chinese	Transfer Care Robot	Transfer assistance	in design
Gu Qifang, 2022	China	Journal articles in Chinese	Bidirectional Transfer Robot	Transfer assistance	in design
Guo Dan, 2013	China	Journal articles in Chinese	Ward Round Robot	Ward round	under test
Guo Shijie et al., 2021	China	Chinese Patent	Nurse Robot	Transfer assistance; Deliver medications and other items	in design

Guo Shijie et al., 2021	China	Chinese Patent	Nurse Robot	Deliver medications and other items	in design
Guo Shijie et al., 2022	China	Chinese Patent	Nursing Robot	Transfer assistance	in design
Guo Shijie et al., 2022	China	Chinese Patent	Dual-arm Robot	Transfer assistance	in design
Guo Shijie et al., 2022	China	Chinese Patent	Nurse Robot	Transfer assistance	in design
Guo Xiao Ya, 2019	China	Journal articles in Chinese	Intelligent Sputum Suction Robot	Expectoration	under test
Han Jinhua et al., 2009	China	Journal articles in Chinese	Nurse Assistant Robot	Deliver medications and other items; Disinfection of wards	in design
Han Zongzhen, 2006	China	Journal articles in Chinese	Nurse Assistant Robot	Deliver medications and other items; Disinfection of wards	in design
Hao Jiyao et al., 2021	China	Chinese Patent	Medical Robot	Deliver medications and other items	in design

Hu Jianjun & Lin Tili, 2017	China	Chinese Patent	Medical Nursing Robot	Transfer assistance	in design
Hu Jianjun et al., 2017	China	Chinese Patent	Nursing Robot	Transfer assistance	in design
Huang Dazhi et al., 2013	China	Journal articles in Chinese	Nurse Assistant Robot	Deliver medications and other items	under test
Humphreys et al., 2023	USA	Journal articles in foreign languages	Advanced Robotic Assist Robot	Transfer assistance	in design
Hung et al., 2021	Canada	Journal articles in foreign languages	Emotional Robot	Psycho-social support	in application
Imamura et al., 2017	Japan	Journal articles in foreign languages	Transfer Support Robot	Transfer assistance	in design

Jiang Haitao, 2011	China	Journal articles in Chinese	Mobile Robot	Ward round	in design
Jiang Yue et al., 2020	China	Chinese Patent	Intelligent Back-Patting and Turnover Robot	Turn over and pat on the back	in design
Jin Haiyang, 2017	China	Chinese Patent	Nurse Assistant Robot	Admission guidance; Health education; Deliver medications and other items	in design
Kato et al., 2022	Japan	Journal articles in foreign languages	Bath Assist Robot	Bath assistance	in application
Kong et al., 2023	Korean	Journal articles in foreign languages	Lift-Assist Robot	Transfer assistance	under test
Li Changhong et al., 2022	China	Chinese Patent	Nursing Lifting Robot	Transfer assistance	in design

Li Fei, 2017	China	Journal articles in Chinese	Nurse Assistant Robot	Deliver medications and other items	in design
Li Hongzhen, 2014	China	Journal articles in Chinese	Wounded Transfer Robot	Transfer assistance	under test
Li Hui & Li Sida, 2017	China	Chinese Patent	Intravenous Infusion Automated Monitoring Robot	Intravenous infusion	in design
Li Min et al., 2021	China	Chinese Patent	Nursing Robot	Transfer assistance	in design
Li Renjun et al., 2021	China	Chinese Patent	Nursing Robot	Deliver medications and other items	in design
Li Rongkuan, 2011	China	Journal articles in Chinese	Ward Round Robot	Ward round	under test
Li Shuangshuang, 2018	China	Journal articles in Chinese	Back Carry Mobility Care Robot	Transfer assistance	under test

Li Shunda, 2019	China	Journal articles in Chinese	Transportation Nursing Robot	Transfer assistance	in design
Li Wenchao et al., 2022	China	Chinese Patent	Body Lift Care Robot	Transfer assistance	in design
Li Wenliang et al., 2022	China	Journal articles in Chinese	Logistics Robot; Intelligent Answering Robot	Admission guidance; Health education; Deliver medications and other items	in application
Li Yan & Li Maoxin, 2022	China	Chinese Patent	Medicine Delivery Robot	Deliver medications and other items	in design
Li Yan, 2021	China	Journal articles in Chinese	Artificial Intelligence Education Robot	Health education	in application
Li Yang et al., 2023	China	Journal articles in Chinese	Dual-arm Robot	Transfer assistance	in design
Li Yantao, 2012	China	Journal articles in Chinese	Meal Assistant Robot	Meal assistance	in design

Li Yong et al., 2020	China	Chinese Patent	Nurse Assistant Robot	Deliver medications and other items	in design
Li Yueqin, 2021	China	Chinese Patent	Medication Care Robot	Deliver medications and other items	in design
Li Yuqing et al., 2008	China	Journal articles in Chinese	Mobile Nursing Robot	Roll-over assistance	in design
Liang Jiale et al., 2020	China	Journal articles in foreign languages	Nursing Robot	Transfer assistance	in design
Liang Xiaobing, 2020	China	Chinese Patent	Roll-over Nursing Cleaning Robot	Roll-over assistance; Urinary and fecal care	in design
Liang Yu & Zeng Ming, 2022	China	Chinese Patent	Fully Intelligent Deployment Robot	Drug configurations for other routes of administration	in design
Lin	China	Journal articles	Dual-arm Transfer Robot	Transfer assistance	under test

Chuan, 2018		in Chinese			
Liu Baochun et al., 2013	China	Chinese Patent	Automated Dispensing Robot	Drug configurations for intravenous infusions	in design
Liu Dong, 2018	China	Chinese Patent	Intelligent Nursing Robot	Transfer assistance	in design
Liu et al., 2021	China	Journal articles in foreign languages	Transfer Robot	Transfer assistance	in design
Liu Yuxin et al., 2022	China	Journal articles in foreign languages	Piggyback Nursing Care Robot	Transfer assistance	in design
Liu Haifeng et al., 2022	China	Chinese Patent	Nursing Robot	Transfer assistance	in design
Liu Haifeng et al., 2022	China	Chinese Patent	Transfer Nursing Robot	Transfer assistance	in design
Liu Han	China	Journal articles	Intravenous Blood Collection Robot	Phlebotomy	under test

Song, 2022		in Chinese			
Liu Hua, 2018	China	Journal articles in Chinese	Medically Assisted Lifting Robot	Transfer assistance	under test
Liu Jinyue et al., 2017	China	Chinese Patent	Transportation Nursing Robot	Transfer assistance	in design
Liu Jinyue et al., 2017	China	Chinese Patent	Transportation Robot	Transfer assistance	in design
Liu Junfei, 2012	China	Journal articles in Chinese	MT-Bear	Transfer assistance	under test
Liu Teng et al., 2022	China	Chinese Patent	Transportation & Carrying Care Robot	Transfer assistance	in design
Liu Yang et al., 2020	China	Journal articles in Chinese	Nursing Robot	Transfer assistance	in design
Liu Yang, 2021	China	Journal articles in Chinese	Nursing Robot	Transfer assistance	in design
Liu Yunyun et al., 2022	China	Chinese Patent	Turnover Care Robot	Roll-over assistance	in design

Lu Hao et al., 2021	China	Chinese Patent	Nurse Robot	Transfer assistance	in design
Lv Xiaoyang, 2011	UK	Journal articles in Chinese	TUGs	Deliver medications and other items	in application
Lv Yi et al., 2016	China	Chinese Patent	Sputum Suction Robot	Expectoration	in design
Lv Yi et al., 2016	China	Chinese Patent	Sputum Suction Robot	Expectoration	in design
Lv Yi et al., 2021	China	Chinese Patent	Intelligent Urinary Catheterization Robot	Urinary catheterization	in design
Ma Jianming, 2017	China	Journal articles in Chinese	Ward Service Robot	Deliver medications and other items; Disinfection of wards	in design
Machi, 2021	China	Chinese Patent	Automatic Medication Delivery Robot	Deliver medications and other items	in design
Matsumoto et al., 2016	Japan	Journal articles in foreign	Transportation Nursing Robot	Transfer assistance	under test

		languages			
Min Xiyao et al., 2021	China	Chinese Patent	Intelligent Drug Delivery Robot	Deliver medications and other items	in design
Narayanan et al., 2022	Vietnam	Journal articles in foreign languages	Autonomous Nursing Robot	Deliver medications and other items; Take blood pressure and pulse; Take body temperature	in design
Nguyen et al., 2013	Japan	Journal articles in foreign languages	Autonomous Navigating Robot	Transfer assistance	in design
No Author, 1999	China	Journal articles in Chinese	Assistant Robot	Transfer assistance	under test
No Author, 2011	Japan	Journal articles in Chinese	RIBA	Transfer assistance	in design
No Author, 201	China	Chinese Patent	Ward Data Capture Robot	Ward round	in design

5					
No Author, 2016	China	Journal articles in Chinese	Intravenous Drug Dispensing Robot	Drug configurations for intravenous infusions	in application
No Author, 2016	China	Journal articles in Chinese	Puncture and Blood Collection Robot	Phlebotomy	in design
No Author, 2020	USA	Journal articles in Chinese	Veebot	Phlebotomy	in application
No Author, 2020	China	Journal articles in Chinese	Blood Collection Robot	Phlebotomy	in design
No Author, 2021	Japan	Journal articles in foreign languages	RIBA	Transfer assistance	in application
Pepito et	Japan	Journal articles	MOXI	Psycho-social support	in application

al., 2020		in foreign languages			
Qi Jincai, 2018	China	Chinese Patent	Intelligent Medical Robot	Transfer assistance	in design
Qianlian Hengmei, 2 019	China	Journal articles in Chinese	Ward Round Robot	Ward round	in design
Qin Tongyun, 20 20	China	Chinese Patent	Nursing Robot	Deliver medications and other items	in design
Sang Lingfeng et al., 2022	China	Journal articles in Chinese	Transfer Robot	Transfer assistance	in design
Sato et al., 2003	Japan	Journal articles in foreign languages	Patrol Robot	Ward round	under test
Shao	China	Chinese Patent	Blood Collection Robot	Phlebotomy	in design

Changnian, 2022					
She Yunjiu, 2008	China	Journal articles in Chinese	Nurse Assistant Robot	Deliver medications and other items; Disinfection of wards	under test
Shen Peng, 2007	China	Journal articles in Chinese	Nurse Assistant Robot	Deliver medications and other items; Disinfection of wards	in design
Song Chunyu et al., 2022	China	Chinese Patent	Intelligent Blood Collection Robot	Phlebotomy	in design
Song Tingting et al., 2021	China	Chinese Patent	Mobile Care Robot	Transfer assistance	in design
Song Yanshu, 2020	China	Journal articles in Chinese	Nursing Robot	Transfer assistance	in design
Su Lin &	China	Chinese Patent	Intelligent Nursing Robot	Deliver medications and other items;	in design

Zhang Xin, 2021				Take blood pressure	
Sun Baoqing et al., 2022	China	Chinese Patent	Intelligent Blood Collection Robot	Phlebotomy	in design
Sun Chunjie et al., 2022	China	Chinese Patent	Drug Transportation Robot	Deliver medications and other items	in design
Sun Yehuan, 2019	China	Journal articles in Chinese	Transportation Nursing Robot	Transfer assistance	in design
Tanioka , 2019	Japan	Journal articles in foreign languages	Emotional Robot	Psycho-social support	in application
Shi Tingqi et al., 2021	China	Chinese Patent	Ward Round Robot	Ward round	in design
Wang Haowen et	China	Journal articles in Chinese	Ward Round Robot	Admission guidance; Ward round; Discharge guidance; Pre-operative	in design

al., 2022				education	
Wang Hongbo et al., 2017	China	Chinese Patent	Bidirectional Transfer Nursing Robot	Transfer assistance	in design
Wang Hongbo et al., 2017	China	Chinese Patent	Transfer Nursing Robot	Transfer assistance	in design
Wang Hongwei et al., 2021	China	Chinese Patent	Medical Mobile Nursing Robot	Deliver medications and other items; Disinfection of wards	in design
Wang Hui et al., 2020	China	Chinese Patent	Intelligent Nursing Robot	Deliver medications and other items; Ward round	in design
Wang Jiachao, 2012	China	Journal articles in Chinese	Ward Round Robot	Ward round	in design
Wang Jian & Xun	China	Chinese Patent	Intelligent Robot	Roll-over assistance	in design

Hao, 2021					
Wang Minhui et al., 2021	China	Chinese Patent	Intelligent Caring and Cleaning Robot	Urinary and fecal care	in design
Wang Shaojiang & Shi Liu, 2021	China	Chinese Patent	Intelligent Nursing Robot	Transfer assistance	in design
Wang Shaokai et al., 2020	China	Chinese Patent	Blood Collection Robot	Phlebotomy	in design
Wang Tao et al., 2019	China	Journal articles in Chinese	Intelligent Intravenous Drug Dispensing Robot	Drug configurations for intravenous infusions	in application
Wang Wenjing et al., 2021	China	Journal articles in Chinese	Intelligent Dispensing Robot	Drug configurations for intravenous infusions	in application
Wang	China	Chinese Patent	Urinary and Fecal Care Robot	Urinary and fecal care	in design

Xiaoqiang, 2021					
Wang Ying, 2016	China	Journal articles in Chinese	Handling Assist Robot	Transfer assistance	in design
Wang Yingqiu & Chen Shidong, 2018	China	Journal articles in Chinese	Infuser Waste Disposal Robot	Intravenous fluid waste disposal (IVFWD)	in design
Wang Zhibo et al., 2019	China	Chinese Patent	Drug Delivery Robot	Deliver medications and other items	in design
Wu Hao et al., 2020	China	Chinese Patent	Nursing Intelligent Health Education Robot	Health education	in design
Wu Yuanchun & Chen Jian, 2018	China	Chinese Patent	Nursing Medicine Delivery Robot	Deliver medications and other items	in design

Xiao Bing et al., 2017	China	Journal articles in Chinese	Medical Round Robot	Ward round; Deliver medications and other items	in design
Xiao Chunhua et al., 2023	China	Chinese Patent	Intelligent Drug Dispensing Robot	Deliver medications and other items	in design
Xiaobo Shi, 2019	China	Journal articles in Chinese	Transportation Care Robot	Transfer assistance	under test
Xu Jibing et al., 2021	China	Chinese Patent	Nursing Robot	Take body temperature; Take blood pressure; Drug configurations for intravenous infusions; Intravenous infusion	in design
Xu Yang, 1997	China	Journal articles in Chinese	Nursing Robot	Deliver medications and other items	in application
Yang Ping et al., 2020	China	Journal articles in Chinese	Intravenous Drug Dispensing Robot	Drug configurations for intravenous infusions	in application
Yang Yang et al., 2021	China	Chinese Patent	Medical Round Medication Delivery Robot	Deliver medications and other items	in design

Yang Yushen & Yang Yuntian, 2006	China	Chinese Patent	Medical Robot	Deliver medications and other items	in design
Yao Chong et al., 2022	China	Journal articles in Chinese	Intelligent Disinfection Robot	Disinfection of wards	in application
Ye Lin, 2021	China	Chinese Patent	Intravenous Infusion Automatic Hanging Medicine Robot	Intravenous infusion	in design
Ying Yi Yun, 2022	China	Chinese Patent	Transfer Robot	Transfer assistance	in design
Yu Changgeng & Yang Chongjun, 2018	China	Chinese Patent	Nursing Service Robot	Deliver medications and other items; Psycho-social support	in design
Yuan	China	Journal articles	Intelligent Sputum Suction Robot	Expectoration	under test

Lirong et al., 2020		in Chinese			
Yuan Su, 2022	China	Chinese Patent	Intelligent Nursing Robot	Intravenous infusion	in design
Yuan Su, 2022	China	Chinese Patent	Intelligent Nursing Robot	Intravenous infusion	in design
Yuan Su, 2022	China	Chinese Patent	Intelligent Nursing Robot	Intravenous infusion	in design
Yun Jintian et al., 2016	China	Journal articles in Chinese	Mobile Humanoid Nursing Robot	Transfer assistance	under test
Zhan Jing et al., 2020	China	Chinese Patent	Intelligent Nursing Robot	Roll-over assistance	in design
Zhang Azhen, 2022	China	Journal articles in Chinese	Little Nurse Robot	Drug configurations for other routes of administration	in design
Zhang Guanjing et al., 2018	China	Chinese Patent	Intravenous Infusion Alarm Robot	Deliver medications and other items	in design

Zhang Jie et al., 2019	China	Chinese Patent	Transfer Robot	Transfer assistance	in design
Zhang Kai, 2013	China	Journal articles in Chinese	Ward Round Robot	Ward round	under test
Zhang Longlong & Xu Longfei, 2021	China	Chinese Patent	Transfer Robot	Deliver medications and other items	in design
Zhang Qing & Chen Shidong, 2018	China	Journal articles in Chinese	Intravenous Infusion-assisted Fluid Change Robot	Replacement of infusion vials	in design
Zhang Tonghe et al., 2020	China	Chinese Patent	Transport Robot	Transfer assistance	in design
Zhang	China	Chinese Patent	Transfer Robot	Transfer assistance	in design

Xizheng et al., 2010					
Zhang Yaoqi et al., 2023	China	Chinese Patent	Ward Round Robot	Ward round; Take body temperature	in design
Zhang Yefei, 2021	China	Chinese Patent	Nursing Robot	Ward round	in design
Zhang Zhijun et al., 2022	China	Chinese Patent	Nursing Robot	Take body temperature	in design
Zhang Zhijun et al., 2022	China	Chinese Patent	Nursing Robot	Take blood pressure and pulse	in design
Zhang Zhijun et al., 2022	China	Chinese Patent	Medical Nursing Robots	Take body temperature; Take blood pressure and pulse; Deliver medications and other items	in design
Zhang	China	Chinese Patent	Nursing Robot	Take body temperature; Take blood	in design

Zhijun et al., 2022				pressure and pulse; Deliver medications and other items	
Zhang Zhijun et al., 2022	China	Chinese Patent	Medical Robot	Ward round	in design
Zhao Lina et al., 2021	China	Chinese Patent	Intelligent Electromagnetic Therapy Robot	Electromagnetic therapy	in design
Zhao Yan, 2018	China	Journal articles in Chinese	Intravenous Dispensing Robot	Drug configurations for intravenous infusions	in application
Zhao Yanzhi et al., 2020	China	Chinese Patent	Transfer Nursing Robot	Transfer assistance	in design
Zhao Yanzhi et al., 2020	China	Chinese Patent	Transfer Nursing Robot	Transfer assistance	in design
Zhao Yanzhi et	China	Chinese Patent	Transfer Nursing Robot	Transfer assistance	in design

al., 2021					
Zhong Ming et al., 2021	China	Chinese Patent	Transfer Robot	Transfer assistance	in design
Zhou Hongzhen et al., 2017	China	Journal articles in Chinese	Intelligent Intravenous Medication Dispensing Robot	Drug configurations for intravenous infusions	in application
Zhou Luhan, 2017	China	Journal articles in Chinese	Medical Robotic	Drug configurations for intravenous infusions	in application
Zhou Meng, 2012	China	Journal articles in Chinese	Ward Round Robot	Ward round	in application
Zhou Yingzhen et al., 2014	China	Journal articles in Chinese	Drug Dispensing Robot	Drug configurations for intravenous infusions	in application
Zhou Zu ming, 2012	China	Journal articles in Chinese	Intelligent Nursing Robot	Roll-over assistance; Urinary and fecal care	under test
Zhu Chan et al., 2018	China	Chinese Patent	Nursing Education Robot	Health education	in design

Appendix III. Cross-sectional survey nursing programs

Basic nursing programs

assisting with washing, shaving, oral care, nail care, combing, shampooing, haircut care, foot care, assisting with bathing, assisting with dressing, cleaning and making up bed units, morning care, evening care, disinfection of wards, replacement of bed linens and covers, assisting with meal preparation, assisting with meal ordering, assisting with meals, washing hands before and after meals, pouring boiled water, changing dietary care level cards, urinary and fecal care, assisting with toileting, assisting with turning over, turning over and patting on the back, air mattress use, assisting with bed mobility, assisting with getting out of bed mobility, admission introduction, discharge guidance, psycho-social support, rehabilitation guidance, health education, safety guidance, preoperative education, delivering of medications and other items, assisting with inspections, laying of anesthesia beds, ward rounds, transfer assistance

Specialized nursing programs

preventive care for bedsores, oral administration, rectal administration, drug configurations for other routes of administration, drug configurations for intravenous infusions, intravenous infusion, replacement of infusion vials, intravenous fluid waste disposal (IVFWD), heparin capping, removal of indwelling needles, transfuse blood,

intravenous (drug) administration, infusion pump use, syringe pump use, subcutaneous administration, intradermal administration, iodine intradermal test, intramuscular administration, phlebotomy, indwelling venous puncture, PICC placement, assisting with deep vein placement (CVC/PICC), intravenous access device maintenance, oxygen administration, addition of wetting solution, oxygen replacement, oxygen bag filling, withdrawal of oxygen absorbing devices, deactivate oxygenation, expectoration, nebulized inhalation, mechanically assisted sputum evacuation, replacement of suction trays, urinary catheterization, urinary catheter removal, retained enemas, massive unretained enemas, cleansing enemas, stoma enemas, placement of gastric tubes, removal of gastric tubes, all types of plumbing care, timed change of urinary bag/drainage bags/balls, pouring of drained fluids, bladder irrigation, continuous bladder irrigation, replacement of bladder flushing solution, specimen management, blood glucose management, GI preparation, coronary arteriograms, skin preparation, umbilical care, preoperative preparation, blood preparation/delivery, preoperative medication, handover from surgery, preoperative positioning, intraoperative care, postoperative care, consultation, dressing change, physical cooling, tube removal, wet magnesium sulphate, cadaveric care, nursing assessment, counting intake and exclusion, take blood pressure, take temperature and pulse, take SPO2, take height/weight, take abdominal circumference,

cardiac monitoring, placement of cardiac monitor, cessation of cardiac monitoring, dynamic electrocardiography and blood pressure 2-in-1 test, peripheral blood flow observation, nasal feeding, enteral nutrition, regulating enteral nutrition solution, replacement of enteral nutritional solution, enterostomy care, puncture to obtain arterial blood specimen, tracheal intubation care, assisting with endotracheal intubation, tracheotomy care, continuous dual trocar flushing, intratracheal tube cleaning and sterilization, non-invasive mechanical ventilation management, cerebral function therapy, swallowing function therapy, EEG monitoring use, non-invasive intracranial pressure monitoring, cold therapy, arteriovenous endovascular fistula care, ultrasonic nebulized infiltration therapy, continuous wound irrigation, low-frequency pulsed electrotherapy, medium-frequency pulsed electrotherapy, TDP (baking lamp), hitech light irradiation, red light therapy, infrared therapy, pneumatic pressure therapy, bone traumatology instrument therapy, ultrasonic therapy, microwave therapy, electromagnetic therapy, millimeter wave therapy, computerized intermediate frequency therapy (IFT), radiofrequency electrotherapy (RFET), laser therapy, biofeedback therapy for liver disease, Korean pain therapy, electro-acupuncture (EA), plaster cast care, care for externally fixed pinholes, alcohol dripping, iodine dripping, traction therapy, splint immobilization, VSD flushing (negative pressure flushing), peritoneal dialysis, peritoneal dialysis fluid

exchange, peritoneal balance test, replacement of peritoneal dialysis external short tubing, APD onboarding, APD disembarkation,colonic dialysis,tunnel port care, hemodialysis, hemodialysis item preparation, hemodialysis specimen delivery, hemodialysis transfer care for critically ill patients, continuous renal replacement therapy（CRRT）, tubing fluid configuration, CRRT bedside care, thrombosis prophylaxis care, assisting with peritoneal puncture, peritoneal puncture for fluid extraction, ambulatory glucose monitoring, ambulatory glucose monitoring installation, ambulatory glucose monitoring rounds, ambulatory glucose monitoring removal, enter CGMS value, insulin pump installation, insulin pump rounds, adjusting insulin pumps, replacement of insulin supplies, evacuation of insulin pumps, hypoglycemia management, blood ketone monitoring, podiatry screening, podiatry wound dressing change, complications preventive care, endocrine specialty test, convalescent training, endotracheal drip, colonoscopy preparation, anorectal manometry, intravenous introduction, monitoring pupils, CVP monitoring, anti-tumor drug configuration, atherosclerosis treatment, vaginal administration, renal puncture biopsy, funduscopic examination, tinnitus treatment, intraocular administration, irrigation of tear ducts, nasal rinse, non-invasive cardiac output testing, resuscitation, electrical resuscitation, preparation of bedside first aid items, analgesic pump administration, mammary ductoscopy
